# The effect of gestational diabetes on the fracture risk after pregnancy: a nationwide register-based study

**DOI:** 10.1007/s00592-022-01907-x

**Published:** 2022-06-18

**Authors:** Matias Vaajala, Rasmus Liukkonen, Ilari Kuitunen, Ville Ponkilainen, Ville M. Mattila

**Affiliations:** 1 Faculty of Medicine and Life Sciences, University of Tampere, Tampere, Finland; 2 Department of Pediatrics, Mikkeli Central Hospital, Mikkeli, Finland; 3 Institute of Clinical Medicine and Department of Pediatrics, University of Eastern Finland, Kuopio, Finland; 4 Department of Surgery, Central Finland Central Hospital Nova, Jyväskylä, Finland; 5 Department of Orthopaedics and Traumatology, Tampere University Hospital, Tampere, Finland

**Keywords:** Gestational diabetes, GDM, Fracture risk, Diabetes

## Introduction

Diabetes mellitus (DM) has been reported to increase the risk of fractures due to pathophysiological changes in the bone endocrinology, as well as disorders in glucose metabolism [1]. The prevalence of DM has continued to increase [2], and subsequently it increases the comorbidity burden higher.

In addition to the increasing prevalence of DM, the rates of gestational diabetes mellitus (GDM) have been increasing during previous decade [3]. Furthermore, as women whose pregnancies are complicated with GDM are in increased risk of developing the type 2 DM [4], it might also increase the risk of fractures amongst these patients.

A previous study from United Kingdom reported, that GDM increases the risk of fractures, especially hip fractures [5]. To the best of our knowledge, no other study has examined the risk of fractures after GDM. Therefore, the aim of this study was to examine, if the GDM increases the risk of fractures in fertile-aged women using nationwide registers.

## Materials and methods

In this retrospective register-based study, data from the National Medical Birth Register (MBR) (1.1.2004–31.12.2013) was combined with data from the Care Register for Health Care (1.1.2004–31.12.2018). Both registers are maintained by the Finnish Institute for Health and Welfare. The MBR contains information on pregnancies, delivery statistics, and the perinatal outcomes, including GDM. We included all pregnancies in women aged 15 to 44 recorded in the MBR between 2004 and 2013 that led to birth. In Finland, GDM is diagnosed in second trimester with 75 g oral glucose test. Data from the Care Register for Health Care contained information on all fractures treated at secondary or tertiary level units between 2004 and 2018. International Classification of Diseases tenth revision (ICD-10) codes were used to identify fracture patients. Fractures of the upper extremity, spine and pelvis, and lower extremity were included in the study. The specific ICD-10 codes with definitions for each fracture included in this study are presented in Appendix 1. The dates of the fracture hospitalization periods found in the Care Register for Health Care were used to compare the risk for a woman sustaining a fracture after giving birth. Based on ICD-10 diagnoses, women with diagnosed type 1 DM were excluded. Forming of the study groups is shown in a flowchart in Appendix 2.

The Cox regression model was used to evaluate the risk for fracture in women with gestational diabetes. Women without gestational diabetes formed the control group. The start point for the follow-up was the date of giving birth. The endpoint of the follow-up was the first fracture hospitalization after giving birth, start of the next pregnancy, or the common endpoint of the follow-up, which was 1–5 years after giving birth, based on the chosen length of the follow-up. The model was created separately with one, two-, three-, four-, and five-year follow-up (each follow-up time starting from the delivery) to analyze the risk with different time periods after the pregnancy, as the length of the changes in physiology after GDM remains unknown. The models with different follow-up times were analyzed separately. The results were interpreted with hazard ratios (HR) and adjusted hazard ratios (aHR) with 95% confidence intervals (CI). Proportional hazards assumption in the cox model was tested using Schoenfeld residuals, and the assumption was not violated. Models were adjusted with body mass index (BMI), age and smoking status of the mother during pregnancy. The adjustments were chosen based on known risk factors for fractures.

## Results

**Fig. 1:**
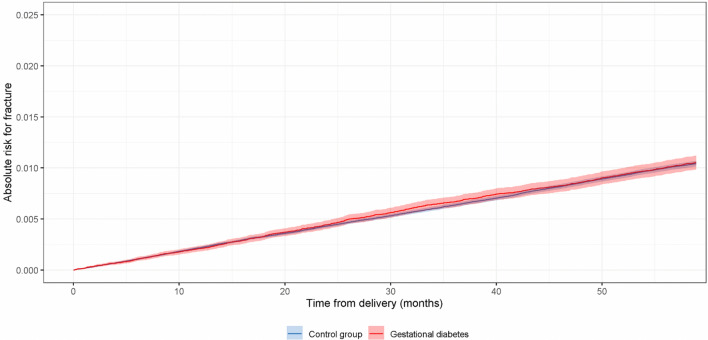
Kaplan–Meier survival curves (with 95% confidence intervals) of women suffering a fracture after giving birth during the following 5-years. Women with gestational diabetes were compared to those without (control group)

A total of 106 146 pregnancies with GDM were included. Of these, 918 (0.86%) women sustained a fracture during the following 5 years after the pregnancy. The control group consisted of 693 338 pregnancies, of which 5494 (0.57%) women sustained a fracture during the following 5 years. Both study groups had similar absolute risk for the fractures after pregnancy throughout the whole 5-year follow-up, the risk increasing up to 0.012 in both groups (Fig. 1).

There was no increased risk for any type of fractures observed among women with GDM. In fact, as the follow-up time lengthened, the risk for fractures was lower among women with GDM. With a 4-year follow-up, the aHR for any fracture for women with GDM was 0.89 (CI 0.82–0.96) and with 5-year follow-up 0.90 (CI 0.83–0.97). Adjusting the models decreased the risk for fractures among women with GMD markedly (Table 1).

**Table 1:** Hazard ratios (HR) and adjusted hazard ratios (aHR) for the event of woman sustaining a fracture of different anatomic regions after giving birth during the follow-up of 1–5 years (each follow-up starting from the delivery). Women with gestational diabetes were compared to those women without. Models were adjusted with smoking status, BMI, and the age of the mother during pregnancy

Follow-up time	1-year	2-year	3-year	4-year	5-year
*Total risk for fractures*					
HR (CI)	0.97 (0.84–1.12)	1.02 (0.92–1.13)	1.05 (0.97–1.15)	1.01 (0.94–1.09)	1.01 (0.95–1.09)
aHR (CI)	0.87 (0.75–1.01)	0.89 (0.80–0.99)	0.92 (0.84–1.01)	0.89 (0.82–0.96)	0.90 (0.83–0.97)
*Risk for upper extremity fractures*					
HR (CI)	0.99 (0.81–1.22)	0.98 (0.85–1.14)	1.01 (0.89–1.15)	0.95 (0.85–1.07)	0.94 (0.85–1.04)
aHR (CI)	0.95 (0.77–1.17)	0.92 (0.79–1.08)	0.96 (0.84–1.09)	0.90 (0.79–1.01)	0.89 (0.80–0.99)
*Risk for fractures of spine or pelvis*					
HR (CI)	0.72 (0.43–1.21)	0.79 (0.54–1.16)	0.75 (0.54–1.04)	0.70 (0.52–0.95)	0.70 (0.53–0.93)
aHR (CI)	0.86 (0.50–1.48)	0.82 (0.55–1.22)	0.77 (0.55–1.08)	0.73 (0.53–0.99)	0.74 (0.55–0.98)
*Risk for lower extremity fractures*					
HR (CI)	1.03 (0.82–1.30)	1.22 (1.04–1.44)	1.27 (1.11–1.45)	1.26 (1.11–1.41)	1.28 (1.15–1.43)
aHR (CI)	0.78 (0.61–1.00)	0.90 (0.76–1.07)	0.94 (0.81–1.08)	0.93 (0.82–1.06)	0.98 (0.87–1.09)

## Discussion

GDM doesn’t increase the risk for fractures after pregnancy based on our results. One study has previously examined the effects of GDM on subsequent fracture risk. In this study, the risk for all fractures and hip fractures was found to be markedly higher [5]. However, based on our results, the toal risk for any fracture after pregnancy was not higher at any case. In addition, the risk for hip fractures was not higher either. According to our results, adjusting the model with BMI, smoking status, and age of the mother decreased the risk for fractures (especially in lower extremity), which might indicate that these factors are more likely the reason for the increase in the risk for fractures, not the physiologic chances caused by GDM.

The strength of our study is the large nationwide register with a GDM registered for all pregnancies during the study period. Furthermore, GDM screening practice has remained unchanged during our study period. The register data used in our study are routinely collected with structured forms with national instructions, which ensures good coverage (over 99%) and reduces possible reporting and selection bias. The main limitation of our study is the missing clinical information on the fractures included in this study (e.g., radiological finding, trauma mechanisms).

## Figures and Tables

**Fig. 2:**
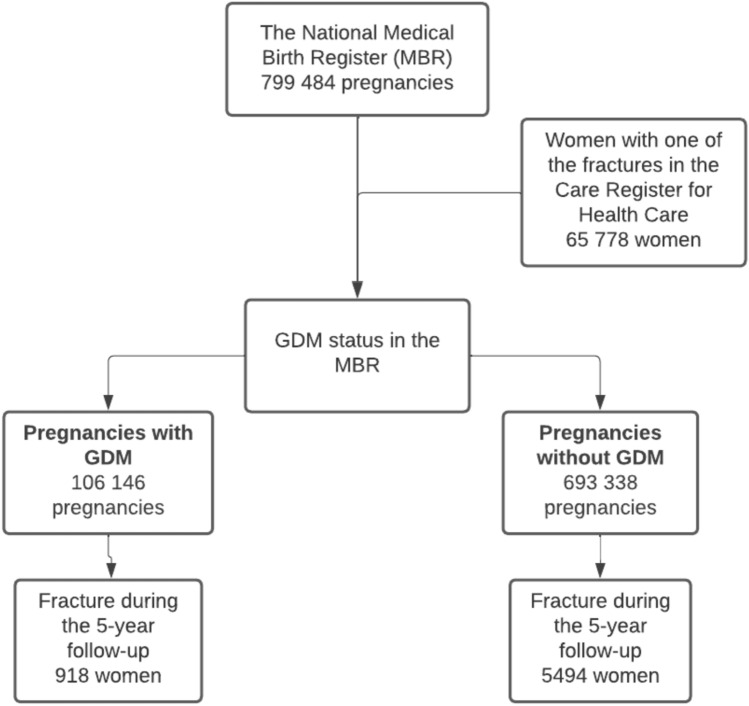
Flowchart of the study population. Data from the Medical Birth Register (MBR) was linked with the diagnosed fractures found in the Care Register for Health Care

## Appendix 1

See Table 2.

**Table 2:** ICD-10 codes with definitions for each fracture included in this study

Fractures of upper extremity ICD-10 code	Definition
S42.0	Fracture of clavicle
S42.1	Fracture of scapula
S42.2	Fracture of upper end of humerus
S42.3	Fracture of shaft of humerus
S42.4	Fracture of lower end of humerus
S42.9	Fracture of shoulder girdle, part unspecified
S52.0	Fracture of upper end of ulna
S52.1	Fracture of upper end of radius
S52.2	Fracture of shaft of ulna
S52.3	Fracture of shaft of radius
S52.5	Fracture of lower end of radius
S52.6	Fracture of lower end of ulna
S52.9	Unspecified fracture of forearm
S62.0	Fracture of navicular bone of wrist
S62.1	Fracture of other and unspecified carpal bone

Fractures of spineor pelvis ICD-10 code	Definition
S12.0	Fracture of first cervical vertebra
S12.1	Fracture of second cervical vertebra
S12.2	Fracture of third cervical vertebra
S12.7	Multiple fractures of cervical vertebra
S12.8	Fracture of other parts of neck
S12.9	Fracture of neck, unspecified
S22.0	Fracture of thoracic vertebra
S22.1	Multiple fractures of thoracic vertebra
S32.0	Fracture of lumbar vertebra
S32.1	Fracture of sacrum
S32.3	Fracture of ilium
S32.4	Fracture of acetabulum
S32.5	Fracture of pubis
S32.7	Multiple fractures of lumbar spine and pelvis
S32.8	Fracture of other parts of pelvis
S32.9	Fracture of unspecified parts of lumbosacral spine and pelvis

Fractures lower extremity subgroup ICD-10 code	Definition
S72.0	Fracture of head and neck of femur
S72.1	Pertrochanteric fracture
S72.3	Fracture of shaft of femur
S72.4	Fracture of lower end of femur
S72.7	Multiple fractures of femur
S72.8	Other fracture of femur
S72.9	Unspecified fracture of femur
S82.0	Fracture of patella
S82.1	Fracture of upper end of tibia
S82.2	Fracture of shaft of tibia
S82.3	Fracture of lower end of tibia
S82.4	Fracture of shaft of fibula
S82.8	Other fractures of lower leg
S82.5	Fracture of medial malleolus
S82.6	Fracture of lateral malleolus
S92.1	Fracture of talus

## Appendix 2

See Fig. 2.
